# Simple multiplex PCR assays to detect common pathogens and associated genes encoding for acquired extended spectrum betalactamases (ESBL) or carbapenemases from surgical site specimens in Vietnam

**DOI:** 10.1186/s12941-015-0079-z

**Published:** 2015-04-12

**Authors:** Ngo Tat Trung, Tran Thi Thu Hien, Tran Thi Thanh Huyen, Dao Thanh Quyen, Mai Thanh Binh, Phan Quoc Hoan, Christian G Meyer, Thirumalaisamy P Velavan, Le Huu Song

**Affiliations:** Department of Molecular Biology, Tran Hung Dao Hospital, No 1, Tran Hung Dao Street, Hai Ba Trung Dist, Hanoi, Vietnam; Department of Gastroenterology, Tran Hung Dao Hospital, No 1, Tran Hung Dao Street, Hai Ba Trung Dist, Hanoi, Vietnam; Institute of Tropical Medicine, University of Tuebingen, Tuebingen, Germany; Fondation Congolaise pour la Recherche Medicale, Brazzaville, Republic of Congo; Institute of Clinical Infectious Diseases, Tran Hung Dao Hospital, No 1, Tran Hung Dao Street, Hai Ba Trung Dist, Hanoi, Vietnam

**Keywords:** Surgical site infection, Betalactam/carbapenem resistance, Vietnam

## Abstract

**Abstract:**

Surgical site infection (SSI) is common in Vietnamese post-operative patients. It contributes to increased morbidity, mortality, hospitalization time and health care expenditure. Bacterial culture is considered the gold standard procedure to identify SSI pathogens and antibiotic resistant properties; however, it can detect microbes that can readily grow and is time-consuming. We propose optimized multiplex PCR assays to diagnose the most relevant microbes and associated genes encoding for acquired extended spectrum betalactamases (ESBL) or carbapenemases from Vietnamese patients with SSI in a hospital setting in Hanoi.

**Methods:**

Ninety-one patients (n = 91) were collected in order to identify microbial pathogens and associated genes encoding for acquired extended spectrum betalactamases (ESBL) or carbapenemases by both conventional bacterial culture and in-house multiplex PCR assays.

**Result and conclusion:**

The novel in-house multiplex PCR assays are comparable to the bacterial culture approach in screening for common pathogens causing SSI and for relevant genotypes conferring betalactam/carbapenem resistance for bacteria. This is the first report of Turkey-specific ESBL gene (PER-1) and two Oxacilinase families (Oxa23 and Oxa 58) in Vietnam.

## Introduction

Hospital-acquired bacterial infection is a common problem in post-operative patients and contributes to increased morbidity, mortality, hospitalization time and health care expenditure [1]. Once surgical site infections (SSI) are detected in patients in medical intensive care, antibacterial/antifungal therapy is implemented. However, the efficacy of antimicrobial treatment largely depends on accurate diagnoses of the microbes causing SSI. To date, bacterial culture is considered the gold standard procedure to identify pathogens that causing SSI. *Staphylococcus aureus, Escherichia coli, Enterococcus* spp.*, Pseudomonas aeruginosa, Enterobacter* spp.*, Candida albicans, Klebsiella pneumonia,* Gram-positive *anaerobes* and *Proteus mirabilis* are the most common causative pathogens of SSI [2]. However, the classical blood culture procedure has two intrinsic problems: (i) it detects only culturable microbes, thus reducing the chance to identify infectious microorganisms from patients treated with antibiotics; (ii) it takes 24 to 48 hours to achieve first test results of cultures, which hampers accurate treatment and risks the patient’s life [3]. Although a broad spectrum of antibiotics is administered post-operatively to control SSI, the increasing occurrence of pathogens resistant to a wide spectrum of antibiotics is alarming, such as meticillin-resistant *S.aureus* (MRSA) or resistance of betalactamases (ESBL)/carbapenemases enterobacteriacea [4,5]. So far, more than one thousand betalactamase genes encoding either ESBL or the carbapenemase phenotype have been recognized [6] (http://www.lahey.org/studies/webt.asp). The distribution of these clinical phenotypes differs greatly between geographical settings and displays distinct local patterns. If conventional bacterial cultures fail to generate colonies, antibiotic resistance profiles cannot be generated.

Vietnam is a tropical country where the risk of infectious diseases remains high. The incidence of SSI detected by bacterial culture reaches up to 33% [7]. Only a few sporadic reports on SSI-related pathogens and no conclusive data on resistance phenotypes are available from Vietnam. Systematic statistics on the betalactamase genotypes that cause epidemics are lacking. Own studies indicate that *Staphylococcus epidermidis, E. coli, Pseudomonas auriginosa, Streptococcus* spp*., Klebsiella pneumonia, Enterobacter* spp*., Staphylococcus aureus and Candida* spp*.* are the most culturable SSI-causative pathogens and we suspect resistance patterns in the genes encoding *VEB, CTX-M of ESBL* group or *NDM-1* of carbapenemase. Here, we introduce simple multiplex PCR assays that provide rapid diagnostic applications and supplement classical bacterial cultures. Furthermore, our multiplexed PCR approach enables to estimate the prevalence of ESBL and/or carbapenem resistance phenotypes in Vietnamese patients with deep incisional SSI.

## Material and methods

### Sampling

Eligible study participants were patients hospitalized at Tran Hung Dao Hospital, Hanoi, Vietnam, between February 2012 and December 2012 with at least one of the following conditions: (1) purulent incisional drainage from deep layers of soft tissue within 30 days of the operation or within 1 year of the operation if a prosthesis was implanted; (2) local signs and symptoms of pain or tenderness, swelling, and erythema, with the incision opened by the surgeon or confirmed by the attending surgeon or physician. Patients provided informed consent and the study was approved by the ethics committee of Tran Hung Dao Hospital. Exudates from deep incisional surgical infection sites (aspirate beneath the incision area) were collected using sterile syringes and, in parallel, subjected to bacterial culture or stored at -80°C for molecular diagnostics. Ninety-one patients (n = 91) were enrolled in the study.

### Bacterial isolates

For optimization of multiplex PCR assays, well-characterized, both biochemically and molecularly, colonies of *Candida albicans, Acinetobacter baumanni, Pseudomonas aeruginosa, Klebsiella pneumoniae, Staphylococcus aureus, Streptococcus pneumoniae, Staphylococcus epidermidis* and *Escherichia coli* were isolated from clinical isolates at the Department of Clinical Microbiology, Tran Hung Dao Hospital, Hanoi, Vietnam.

### Bacterial culture

For the establishment of bacterial cultures we followed the *Manual of Clinical Microbiology* [8]. Briefly, 500 μl aliquots of the purulent incisional drainage from individual biopsies was vortexed well in 800 μl sterilized phosphate buffer saline (PBS). The resulting suspension was streaked onto 4 solid media: sheep blood agar, MacConkey agar, chocolate agar and buffered charcoal-yeast extract agar (BCYE) under aerobic conditions at 37°C for 7 days. Colonial growth was confirmed by medical microbiologists at the Department of Clinical Microbiology, Tran Hung Dao Hospital, Hanoi, Vietnam.

### Design of genus specific primers for multiplex PCR assays to detect microorganisms

**Figure 1:**
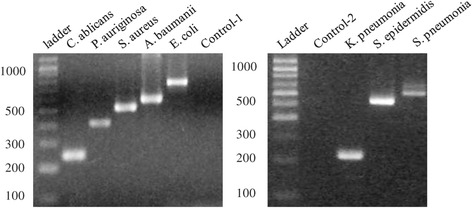
Multiplex PCR assays for screening of SSI associated microorganisms. Left panel MicroSHPT@5leX containing primers specific for *Candida albicans, Acinetobacter baumanni, Pseudomonas aeruginosa, Staphylococcus aureus, Escherichia coli,* whereas MicroSHPT@3leX amplify target genes specific for *Klebsiella pneumonia Streptococcus pneumonia, Staphylococcus epidermidis*. Control-1, control-2 are human whole blood genomics DNA.

To screen for the 8 microbes indicated above, two multiplex PCR assays (MicroSHPT@5leX and MicroSHPT@3leX) were designed such that neighboring amplicons differed by 50 to 100 bps in size, helping to resolve PCR fragments into visible bands in agarose gel electrophoresis (Figure 1).

### Design of family specific primers for multiplex PCR assays to screen for relevant ESBL and carbapenemase encoding genes

To screen for genes of important ESBL and carbapenemases, five multiplex PCR assays, SHPT@ESBL-1(SHV, TEM, CTX-M), SHPT@ESBL-2(VEB, GES, PER), SHPT@Carba-1(NDM, SPM, VIM), SHPT@Carba-2(IMP, AIM, KPC/BIC, DIM), SHPT@Carba-3(Oxa23 like group, Oxa48 like group, Oxa58 like group) were designed. The sizes of neighboring PCR amplicons differed by 50 to 100 bps, again supporting resolution of PCR fragments on agarose gels (Figure 1). Except for the SHPT@Carba-3 (Oxa group) multiplex PCR, all primers target the conserved domain of ESBL gene families (SHV, TEM, CTX-M, VEB, GES, PER)or carbapenemase gene families (NDM, SPM, VIM, IMP, AIM, KPC/BIC, DIM). Primer pairs utilized for screening of genes coding ESBL or carbapenemase are listed in Table 1. The reference strains harboring genetic materials encoding for Oxa48, VIM, SIM, SPM, AIM, IMP, KPC, BIC, DIM were kindly provided by Laurent Poirel [9].

**Table 1:** **Primer sequence used for screening of genes coding ESBL or carbapenemase**

***SHPT@ESBL-1(SHV, TEM, CTX-M)***			***Final concentration (mM)***
590	SHPT108@CTX-M-U1-F	ATGTGCAGYACCAGTAARGTKATGGC	0.4
	SHPT108@CTX-M-U2-R	GGTRAARTARGTSACCAGAAYCAGCGG	0.4
422	SHPT108@TEM-U-F	TCGCCGCATACACTATTCTCAGAATGAC	0.08
	SHPT108@TEM-U-R	CAGCAATAAACCAGCCAGCCGGAAG	0.08
739	SHPT108@TR-SHV-F	TGTATTATCTC(C/T)CTGTTAGCC(A/G)CCCTG	0.48
	SHPT108@TR-SHV-R	GCTCTGCTTTGTTATTCGGGCCAAGC	0.48
***SHPT@ESBL-2(VEB, GES, PER)***			
391	SHPT108@TR-VIM-F	GATGGTGTTTGGTCGCATATCGCAAC	0.08
	SHPT108@TR-VIM-R	CATCGCTGTTGGGGTTGCCCAATTTT	0.08
604	SHPT108@TR-SPM-F	CTGGCAGGGATCGCTCACTC	0.08
	SHPT108@TR-SPM-R	GGTTTCCGATCAGCCACCTCTCA	0.08
731	SHPT108@NDM-1-F primer	CAGTGTGGGGGCCTGACGAT	0.08
	SHPT108@NDM-1-R primer	CTGAGCA ACC TGC GCA ATR ATA GCT T	0.08
***SHPT@Carba-1(NDM, SPM, VIM)***			
320	SHPT108@TR-VIM-F	GATGGTGTTTGGTCGCATATCGCAAC	0.04
	SHPT108@TR-VIM-R	CGAATGCGCAGCACCAGGATAGAA	0.04
291	SHPT108@TR-SPM-F	CGTTTGAAAATCTGGGTACGCAAACG	0.04
	SHPT108@TR-SPM-R	GTTTCAAATCAAAAACATTATCCGCTGGAACAG	0.04
200	SHPT108@NDM-1-F primer	CGAAAGTCAGGCTGTGTTGCGC	0.08
	SHPT108@NDM-1-R primer	GACCGCCCAGATCCTCAACTG	0.08
***SHPT@Carba-2(IMP, AIM, KPC/BIC, DIM)***			
710	SHPT108@Tr-DIM-F	TATTCAGCTTGTCTTCGCTTGCTAACG	0.08
	SHPT108@Tr-DIM-R	GTTAGCGTTCGGCTGGATTGATTTG	0.08
412	TR-KPC-BIC-F	GCTTTCT(T/G)GCTG(C/G)CGC(T/C)GTGCT	0.2
	TR-KPC-BIC-R	AGCCAATCAAC(A/C)A(A/G)CTGCTG(C/A)CGC	0.2
326	SHPT108@AIM-F	CCCTGAAGGTGTACGGAAACAC	0.04
	SHPT108@AIM-R	GGGTTCGGCCACCTCGAATTG	0.04
204	Tr-IMP-F	AC(G/A)GG(C/G/T)GGAATAGAGTGGCTTAA(T/C)TCTC	0.02
	TR-IMP-R	TTCAGG(C/T)A(A/G)CCAAACYACTASGTTATCT	0.02
***SHPT@Carba-3(Oxa23 like group, Oxa48 like group, Oxa58 like group)***			
599	SHPT108@Oxa-58-F	CCCCTCTGCGCTCTACATACAACATC	0.08
	SHPT108@Oxa-58-R	AAGTATTGGGGCTTGTGCTGAGCATAG	0.08
482	Tr-OXA-G23-F2	AGAATATGT(G/C)CC(A/T)GC(C/A)TC(T/A)ACATTTAA(A/G)ATG	0.2
	TR-OXA-G23-R2	CCCA(G/A)CC(G/T)GT(C/T)AACCA(G/A)CC	0.2
286b	Tr-Oxa-G48-F2	CACCAAGTCTTTAAGTGGGATGGACA	0.08
	Tr-Oxa-G48-R2	CCGATACGTGTAACTTATTGTGATACAGCTT	0.08

### DNA extraction and multiplex PCR

An aliquot of the purulent incisional drainage from individual biopsies was streaked onto solid medium for total bacterial culturing; the remnant was immersed into 300 μl universal lysis solution (200 mM NaOH, 1% SDS) and heated for 5 minutes at 95°C. An equal volume of 1 M Tris-HCl was added to neutralize the pH to 7.5. This aqueous solution was subjected to standard phenol/chloroform/isoamyl alcohol extraction [10]. Briefly, 600 μl of aqueous solutionwere transferred into a 1.5 ml Eppendorf tube, an equal volume of phenol/chloroform/isoamyl (24/25/1) was added and vortexed for 5 minutes and centrifuged at 13,000 g. The upper aqueous phase was then transferred to an Eppendorf tube and an equal volume of isopropanol was added. After thorough mixing, the solution was centrifuged at 16,000 g for 30 minutes to pellet the DNA. Precipitated DNA was washed twice with 70% ethanol and reconstituted in 150 μl TE (25 mM Tris-base pH8.0, 1 mM EDTA) and used as template for multiplex PCR in 25 μl reactions containing 10 mM Tris-HCl pH 8.3/50 mM KCl/1.5 mM Mg2^+^, 250 μM of each deoxynucleotide triphosphate and variable amounts of individual primers (Tables 1, and 2). Thermal cycling comprised initial denaturation at 95°C for 4 minutes, 35 cycles of 94°C for 25 seconds, 58°C for 45 seconds and 72°C for one minute. In case the multiplex PCR assays, the amplified products were purified and subjected to PCR sequencing. The resulting sequences reading were compared to gene sequences as described in the NCBI database (Blast search http://blast.ncbi.nlm.nih.gov/Blast.cgi).

**Table 2:** **Target genes and primer sequences used for screeningof infectious microorganisms**

**Pathogens**	**Accession number**	**Forward/Reverse Primer (5′ to 3′)**	**Size (bp)**	**Final concentration (mM)**
C*. albicans*	Z48339	GTGGGTGGTAAATTCCATCTAAAGCTA	243	0.2
		CCGTGCCACATTCCTCCGC		0.2
P. *aeruginosa*	AF116258	CCCGAATGTCGGCATCATTCTC	411	0.2
		CGGTAGACCTCGCGCTTGAA		0.2
S. *aureus*	STAAROA	AAGGGCGAAATAGAAGTGCCGG	515	0.2
		ATGGTCGGTTCCTTAGAAAACAAACTTG		.02
A. *baumannii*	JX470958	TTGGGGCCTTTGAGGCTTTAGTG	599	0.2
		TGGTGCAACAAACTCCCATGGT		0.2
E. *coli*	S-uidA	GTCGCGAGTGAAGATCCCTTTC	773	0.2
		GCATTAATGGACTGGATTGGGGC		0.2
K. *pneumoniae*	Kp-aldA	CCTTGTCTTTAAACGCGCGC	332	0.2
		TTTTTCGCCGCAGCGG		0.2
S. *epidermidis*	AF298800	CGGTATCTTAGTTGTATCTGCTGC	637	0.2
		AGATAATACGTATACTTCAGCTTTGAATTTTGTG		0.2
S. *pneumoniae*	Sp LytA	CAACCGTACAGAATGAAGCGGATTAT	701	0.2
		GTCCTTGTACTTGACCCAGCCT		0.2

## Results

### Multiplex PCR assays enhance positive rate of SSI

After optimizing amplifying conditions for MicroSHPT@5leX and MicroSHPT@3leX (Figure 1, Table 2), the multiplex PCR assays were applied to screen for infected pathogens from 91 SSIs. Fourty specimens tested positive for colonies of at least one strain and 60 out of the 91 SSI specimens were confirmed as bacterial infections by multiplex PCR assays (Table 3). With the exception of *Citrobacter* spp*.*, all positive colonies of any SSIs specimen were confirmed by either MicroSHPT@5leX or MicroSHPT@3leX reactions. Four samples were negative by bacterial culture but positive by multiplex PCR assays. Bacterial culture detected fewer infected samples than did the multiplex PCR assays. This clearly demonstrates that our multiplex PCR assays are more sensitive and accurate in detecting SSI than bacterial cultures.

**Table 3:** **Positive rates of Vietnamese SSI specimens detected by multiplex PCR assays and bacterial cultures**

**Bacterial strain**	**Bacterial culture**	**Multiplex PCR**
*Candida albicans*	0	0
*Acinetobacter baumanni*	4	12
*Pseudomonas aeruginosa*	9	18
*Klebsiella pneumonia*	5	4
*Staphylococcus aureus*	12	13
*Streptococcus pneumonia*	0	0
*Staphylococcus epidermidis*	3	3
*Escherichia coli*	6	10
*Citrobacter sp*	1	0
***Total (positive)***	**40**	**60**
***Total patient samples***	**91**	**91**

### Genotypic prevalence of betalactamases detected from SSI specimens

**Figure 2:**
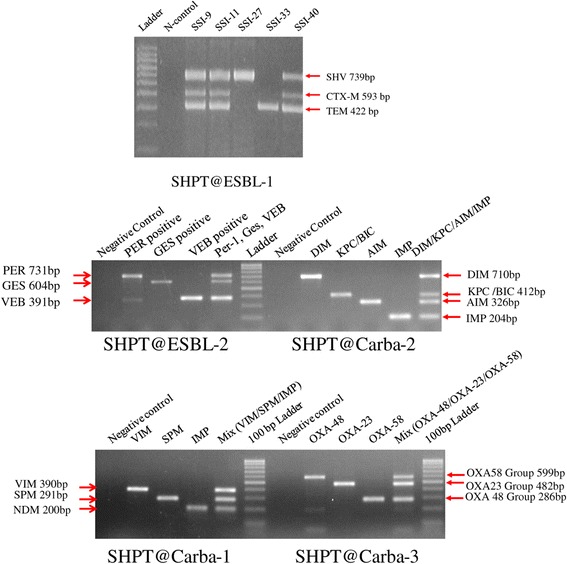
Multiplex PCR assays for screening of acquired ESBL and carbapenemase encoding genes from SSI specimens. Upper left panel is SHPT@ESBL-1 assay to detect SHV, TEM, CTX-M genes; upper right panel is SHPT@ESBL-2 assay to detect VEB, GES, PER genes; lower left panel is SHPT@Carba-1 to screen for NDM, SPM, VIM and SHPT@Carba-2 to screen for IMP, AIM, KPC/BIC, DIM; lower right panel is SHPT@Carba-3 to screen for Oxa23 like group, Oxa48 like group, Oxa58 like group; control samples are human whole blood genomics DNA.

After optimizing the amplification conditions for SHPT@ESBL-1, SHPT@ESBL-2, SHPT@Carba-1, SHPT@Carba-2, SHPT@Carba-3 assays (Figure 2), the five multiplex PCR reactions were used to screen for potential ESBL or cabapenemase genotypes from all 60 PCR-positive SSI samples (Table 3). The screening revealed SHV as the most common ESBL gene family; SHV genotypes existed in 10 (16.6%) of 60 SSI PCR-positive samples. Other ESBL gene families such as *CTX-M* or *TEM* were also detected in a considerable number of biopsies (Table 3). Especially, five of 60 SSI PCR-positive samples (NKSM9, NKSM11, NKSM28, NKSM33, NKSM40) were positive for carbapenemase *NDM-1*; and also had either SHV or CTX-M or TEM. One sample (SSI28) had Turkey-specific ESBL gene (*PER-1)* and oxacilinase gene families (*Oxa23* and *Oxa 58)*.

## Discussion

Hospital acquired bacterial infections after surgical interventions and antibiotic resistances are a major public health threat. Here, we report two optimized multiplex PCR assays that can detect genetics materials of eight most common microbial pathogens, namely *Candida albicans, Acinetobacter baumanni, Pseudomonas aeruginosa, Klebsiella pneumoniae, Staphylococcus aureus, Streptococcus pneumoniae, Staphylococcus epidermidis* and *Escherichia coli.* The assays have detection limits ranging from 10 to 50 CFU/ml human blood (unpublished data). The bacterial identification capability of our multiplex PCR is comparable to that of the conventional blood culture approach, especially in some patient biopsies. PCR was more sensitive than blood cultures.

Antibiotic resistance related to nosocomial infections is of increasing concern and requires the development of suitable tracking tools. Clinical experience in our institution has led us to suspect the existence of microorganisms with extended-spectrum betalactamase or carbapenemase capability. However, under antibiotic pressure in the course of surgical prophylaxis some bacteria cannot proliferate. To overcome this challenge, we have optimized relevant multiplex PCR assays to track for Vietnam-specific ESBL such as VEB-1 [11], blaCTX-M-15, blaCTX-M-27 genes [12] or other dangerous ESBL genes such as NDM-1, OXA48, GIM-1. Our data reveal that, despite the lack of colony growth from patient samples NKSM28, NKSM54 (Table 4), our PCR assays could detect the bacterial genetic material and relevant betalactamase encoding genes (NDM-1, SHV, CTX-M, GIM-1, VEB, OXA-58 in patient sample NKSM28, or SHV in NKSM54 - Table 4).

**Table 4:** **Genotypic prevalence of betalactamases detected from SSI specimens**

	**Bacterial strain bearing the betalactamse genes**		**Detected betalactamse genes by multiplex PCR assays**								
**SSI ID**	**Multiplex CR**	**Bacterial culture**	**NDM 1**	**SHV**	**CTX-M**	**TEM**	**OX23**	**Per-1**	**GIM-1**	**VEB**	**OXA-58**
NKSM9	*E. coli, K. pneumonia, A. baumanii*	*E. coli*	Positive	Positive	Positive	Positive					
NKSM11	*E coli*	*E coli*	Positive	Positive	Positive	Positive					
NKSM28	*A. baumanii*	*Negative*	Positive	Positive	Positive	Positive			Positive	Positive	Positive
NKSM33	*E. coli, K. pneumonia, A. baumanii, P. aurigninosa*	*E. coli, P. aurigninosa*	Positive	Positive	Positive	Positive					
NKSM40	*K. Pneumonia*	*K. Pneumonia*	Positive	Positive	Positive	Positive					
NKSM49	*P. aurigninosa*	*P. aurigninosa*		Positive							
NKSM53	*P. aurigninosa, S. aureus, Ecoli*	*Ecoli*		Positive		Positive					
NKSM54	*A. baumanii*	*Negative*		Positive							
NKSM55	*P. aurigninosa*	*P. aurigninosa*		Positive							
NKSM91	*A. baumanii*	*A. baumanii*					Positive	Positive			Positive
**Total n(%)**			**5 (5)**	**9(9)**	**5(5)**	**6(6)**	**1(1)**	**1(1)**	**1(1)**		**2(2)**

Although PCR based assays are much sensitive and can detect microbes causing SSI, conventional blood culture procedures should not be ignored for obvious reasons. In addition, PCR based assays alone will not allow to get a comprehensive profile SSI related pathogens or SSI associated antibiotic resistance profiles. Therefore, both methodologies should be considered which shall provide vital information on appropriate administration of drugs during SSI.

## Conclusion

In summary, we developed simple optimized multiplex PCR assays that clearly enhance the positive diagnostic rate of deep incisonal SSI compared to the conventional bacterial culture approach. Our multiplex PCR assays also quickly detected important associated genotypes that confer betalactam resistance for bacteria. Also this is the first report of Turkey-specific ESBL gene (*PER-1*) and two Oxacllinase families (*Oxa23* and *Oxa 58*) in Vietnam.
